# Health facility delivery service utilization and its associated factors among women in the pastoralist regions of Ethiopia: A systematic review and meta‐analysis

**DOI:** 10.1002/hsr2.1183

**Published:** 2023-03-29

**Authors:** Anteneh Mengist Dessie, Denekew Tenaw Anley, Melkamu Aderajew Zemene, Eden Workneh Aychew, Habtamu Geremew Debebe, Natnael Moges Misganaw, Chalachew Yenew Denku, Tiruayehu Getinet Abebe, Asaye Alamneh Gebeyehu, Desalegn Tesfa Asnakew, Rahel Mulatie Anteneh, Sefineh Fenta Feleke

**Affiliations:** ^1^ Department of Public Health, College of Health Science Debre Tabor University Debre Tabor Ethiopia; ^2^ Department of Midwifery, College of Health Science Debre Tabor University Debre Tabor Ethiopia; ^3^ College of Health Science Oda Bultum University Chiro Ethiopia; ^4^ Department of Pediatrics and Child Health Nursing, College of Health Science Debre Tabor University Debre Tabor Ethiopia; ^5^ Department of Public Health, College of Health Science Woldia University Woldia Ethiopia

**Keywords:** associated factors, Ethiopia, health facility delivery, pastoralist regions

## Abstract

**Background and Aims:**

Utilizing health facility delivery services is one of the pillars of lowering maternal mortality. However, the coverage of health facility delivery service utilization continues to be uneven around the world. In Ethiopia, particularly among pastoralist regions, health facility delivery service utilization is less common. Therefore, the purpose of this study was to determine the pooled prevalence of health facility delivery service utilization and identify the associated factors among women in the pastoralist regions of Ethiopia.

**Methods:**

A comprehensive systematic search was carried out in PubMed/MEDLINE, Hinary, Cochrane Library, Google Scholar, Google, and Ethiopian online university repositories. Studies were appraised using the JBI appraisal checklist. The analysis was done using STATA version 16. The pooled analysis was conducted using DerSimonian and Laird random‐effects model. *I* ^2^ test and Eggers & Begg's tests were used to assess the heterogeneity and publication bias, respectively. *p* < 0.05 was set to determine the statistical significance of all the tests.

**Results:**

The pooled prevalence of health facility delivery service utilization was 23.09% (95% CI: 18.05%−28.12%). Have ANC visit during pregnancy (OR = 3.75, [95% CI: 1.84−7.63]), have information regarding maternal health service fee exemption (OR = 9.51, [95% CI: 1.41−64.26]), have a nearby health facility (OR = 3.49, [95% CI: 1.48−8.20]), and women attend secondary and above education (OR = 3.06, [95% CI: 1.77−5.29]) were found to be significant associated factors.

**Conclusions:**

Health facility delivery service utilization is very low in pastoralist regions of Ethiopia, and ANC follow‐up, distance from the health facility, women's educational status, and information regarding maternal health service fees were identified as significant associated factors. Consequently, strengthening ANC services, introducing free health services to the community, and constructing health facilities for the nearby residents are recommended to improve the practice.

## 1 BACKGROUND

Around 303,000 women pass away worldwide each year as a result of pregnancy‐related or delivery problems.^1^ Approximately 86% (254,000) of the estimated global maternal death in 2017 occurred in Sub‐Saharan Africa (SSA) and Southern Asia. Approximately two‐thirds (196,000) of all maternal death worldwide occurred in SSA alone.^2^ With 13,000 maternal deaths annually, Ethiopia ranks fourth in terms of maternal mortality burden.^1^ The high rate of maternal mortality in various regions of the world is a reflection of unequal access to high‐quality healthcare services and highlights the gap between rich and poor.

The sustainable development goal aimed to reduce the global maternal mortality ratio (MMR) to below 70, with no country having more than 140 MMR per 100,000 births.^2^ Thus, since the majority of maternal deaths and obstetric problems cluster around the time of delivery^3^ and cannot be predicted a priori, one of the cornerstones to reducing maternal mortality is health facility delivery, where births are aided by trained medical personnel. For lowering maternal and newborn deaths, it is crucial to guarantee that skilled medical workers assist with every birth. Skilled attendance during labor, delivery, and the first several weeks after childbirth could reduce the number of maternal deaths by 13%−33%.^4^ In 2020, 83% of births were attended by skilled medical personnel, but coverage is still uneven worldwide with big regional variations.^5^ Almost 60% of African women give birth at home without the assistance of trained birth attendants, compared to less than 1% in developed nations.^6^

In Ethiopia, the prevalence of health facility delivery service utilization varies between regions and location.^7^, ^8^, ^9^ According to the 2016 Ethiopian Demographic and Health Survey (EDHS), the national prevalence of home delivery was 73%, with considerable regional variances ranging from 3% in Addis Ababa to 85% in Afar.^7^ In more than 122 districts throughout Ethiopia, pastoralist regions occupy 61% of the total area.^10^ According to studies, Ethiopia's pastoralist groups have a higher proportion of home deliveries than other populations. For instance, research in pastoralist areas of Dubti, Afar Region, Ethiopia revealed that 92.6% of births occurred at home.^11^ Similar to this, 83.3% of deliveries were made at home as reported in the semi‐pastoralist village of Malie District, Southern Ethiopia.^12^

Despite the importance of giving birth in a health facility, pastoral women in Ethiopia use the service at a very low level. Therefore, it is imperious to determine the pooled prevalence and investigate factors associated with utilization of health facility delivery service among women in pastoralist regions of Ethiopia. Decision‐makers and program managers might use it as a gate to design evidence‐based strategies to overcome barriers hindering predominantly pastoralist women from giving birth in health facilities.

## 2 METHODS

### 2.1 Study protocol registration

This systematic review and meta‐analysis was conducted to estimate the pooled prevalence of health facility delivery service utilization and identify the associated factors among women in pastoralist regions of Ethiopia at their last childbirth. The Preferred Reporting Items for Systematic Reviews and Meta‐Analyses Protocol (PRISMA‐P) 2015 statement served as the basis for the development of the protocol for this systematic review and meta‐analysis^13^ and the protocol has been registered in PROSPERO‐International Prospective Register of Systematic Reviews (CRD42022300210).

### 2.2 Search strategy

A comprehensive systematic search for all relevant studies was carried‐out in PubMed/MEDLINE, Hinary, Cochrane Library, Google Scholar, and Google. Besides, a search of online university repositories (University of Gondar and Addis Ababa University) and the reference list of already identified studies to retrieve additional articles were done. Throughout the comprehensive literature search, the following search terms were used: “institutional delivery” OR “skilled delivery” OR “health facility delivery” OR “home delivery” OR “home birth” OR “giving birth at home” AND “associated factors” OR “determinants” OR “predictors” AND “women” OR “mother” AND “pastoralists” OR “pastoral community” AND “Ethiopia.” Boolean operators “AND” and “OR” were used to combine the search terms as appropriate. The PRISMA standards were followed in the selection of publications, data extraction, and reporting of results for the review.^14^

### 2.3 Study selection and eligibility criteria

Every article that was found by the search approach was exported to Endnote version 7 to remove duplicate studies. Two sets of reviewers independently examined titles and abstracts for inclusion in full‐text evaluation after duplicate papers were deleted. The defined criteria for choosing articles determined the resolution of the reviewers' disagreement.

#### 2.3.1 Inclusion criteria

all observational studies (cross‐sectional, case controls, and cohort) those reporting the prevalence and/or associated factors; studies among women in pastoralist regions of Ethiopia; literatures written in English language; and both published and unpublished studies at any time were included.

#### 2.3.2 Exclusion criteria

Studies that are unavailable because they have not been published, are not retrievable from the internet, or have not had an email response from the respective authors have been omitted. After a full text examination, a study that did not provide enough information to determine the outcome of interest, non‐full text papers, and secondary studies were also excluded from this analysis.

### 2.4 Quality assessment and critical appraisal

The qualities of each article selected for inclusion in the systematic review (i.e., those that meet the inclusion criteria described above) were assessed by using the Joanna Briggs Institute (JBI) quality appraisal checklist adapted for cross‐sectional, case‐control, and cohort studies.^15^ The assessment was done independently by two set of reviewers to assess the methodological quality of a study and to determine the extent to which a study has addressed the possibility of bias in its design, conduct, and analysis. The third reviewer dealt with any disagreements, and the reviewers discussed their differences until a consensus was established. Studies considered low risk whenever fitted to 50% and or above quality assessment score.^16^

### 2.5 Data extraction

A clear data extraction format was prepared by the principal author in Microsoft Excel. It included names of author, year of publication, the target population, the study region, study design, sample size, response rate, prevalence, number of success and failure in exposed and unexposed groups, and crude odds ratio with confidence Interval (CI) of associated factors. Data was extracted using this structured data extraction form by two separate sets of reviewers. The phase was repeated whenever differences in the extracted data were observed. As long as there were disagreements amongst the data extractors, a third reviewer was brought in, and the reviewers talked things out until they all agreed.

### 2.6 Data synthesis and analysis

The extracted data in Microsoft Excel were imported to STATA version 16 for analysis. Pooled analysis was conducted using a DerSimonian and Laird random‐effects model which assumes heterogeneity across studies.^17^, ^18^ *I* ^2^ test was used to check the heterogeneity of included studies. The values of 25%, 50%, and 75% were declared as low, moderate, and high heterogeneity, respectively.^19^ With the evidence of heterogeneity, subgroup analysis was computed by considering study region as a grouping variable to further explore it. In addition, the sensitivity analysis was also performed using both metaninf and metaplot command to assess whether the pooled prevalence estimates were affected by single studies. Publication bias across studies was also checked using funnel plot and more objectively through Eggers and Begg's tests. Trim and fill analysis was conducted to overcome the publication bias. The effect of selected determinant variables was analyzed using separate categories of meta‐analysis. The findings of the meta‐analysis were presented using forest plot and odds ratio (OR) with its 95% CI. All the tests were two‐sided, and a *p* < 0.05 was set to determine the statistical significance of the tests.

## 3 RESULTS

### 3.1 Characteristics of the included studies

**Figure 1:**
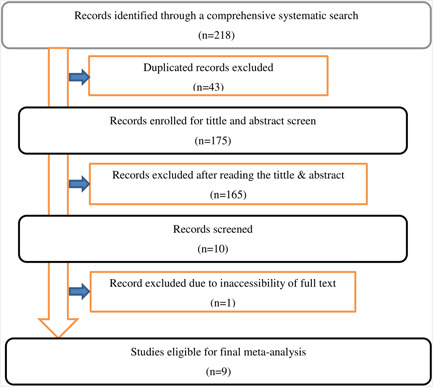
Flow chart of the study selection process for this systematic review and meta‐analysis.

A total of 218 articles were retrieved using a search strategy about health facility delivery service utilization and associated factors among women in pastoralist regions of Ethiopia. Of these, 43 duplicated records were deleted and 175 articles were excluded by the screening of titles and abstracts. Therefore, a total of 10 articles assessed for eligibility on the basis of the inclusion and exclusion criteria, resulting further exclusion of 1 article due to inaccessibility of full text (Figure 1). As a result, 9 articles were met the inclusion criteria to undergo the final systematic review and meta‐analysis (Table 1).

**Table 1:** Characteristics of included studies in this systematic review and meta‐analysis.

Authors	Publication year	Study design	Study region	Sample size	Prevalence (%)	Quality
Assefa et al.^20^	2018	CBCS	Afar	423	35.2	Low risk
Sadik et al.^21^	2019	CBCS	Afar	383	35.2	Low risk
Mekonnen et al.^22^	2012	CBCS	Afar	502	16.7	Low risk
Wondimu and Woldesemayat^23^	2020	CBCC	SNNP	309	NA	Low risk
Wako and Kassa^24^	2017	CBCS	Oromia	876	13.9	Low risk
Zepro and Ahmed^25^	2016	CBCS	Somali	385	30.4	Low risk
Dejene and Hailemariam^12^	2015	CBCS	SNNP	756	14.5	Low risk
Ahmed et al.^26^	2018	CBCS	Afar	2009	18.4	Low risk
Umer et al.^27^	2020	CBCS	Somali	450	22.6	Low risk

Abbreviations: CBCC, community based case control; CBCS, community based cross‐sectional.

### 3.2 Prevalence of health facility delivery service utilization

**Figure 2:**
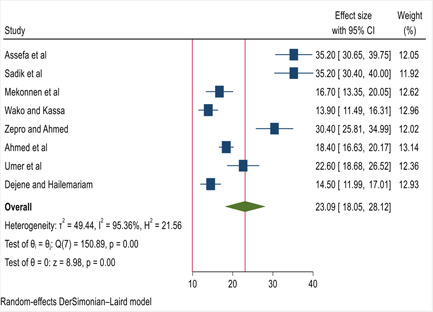
Forest plot for the pooled prevalence of health facility delivery service utilization among women in pastoralist regions of Ethiopia.

One case‐control study was excluded on the prevalence estimation (Wondimu & Woldesemayat).^23^ Then, using a DerSimonian and Laird random‐effects model, the overall pooled prevalence of health facility delivery service utilization among women in pastoralist regions of Ethiopia was 23.09% (95% CI: 18.05%−28.12%) with significant heterogeneity between studies (*I* ^2^ = 95.36%, *p* < 0.001). The overall pooled prevalence of health facility delivery service utilization was presented using a forest plot (Figure 2).

### 3.3 Heterogeneity and publication bias

**Figure 3:**
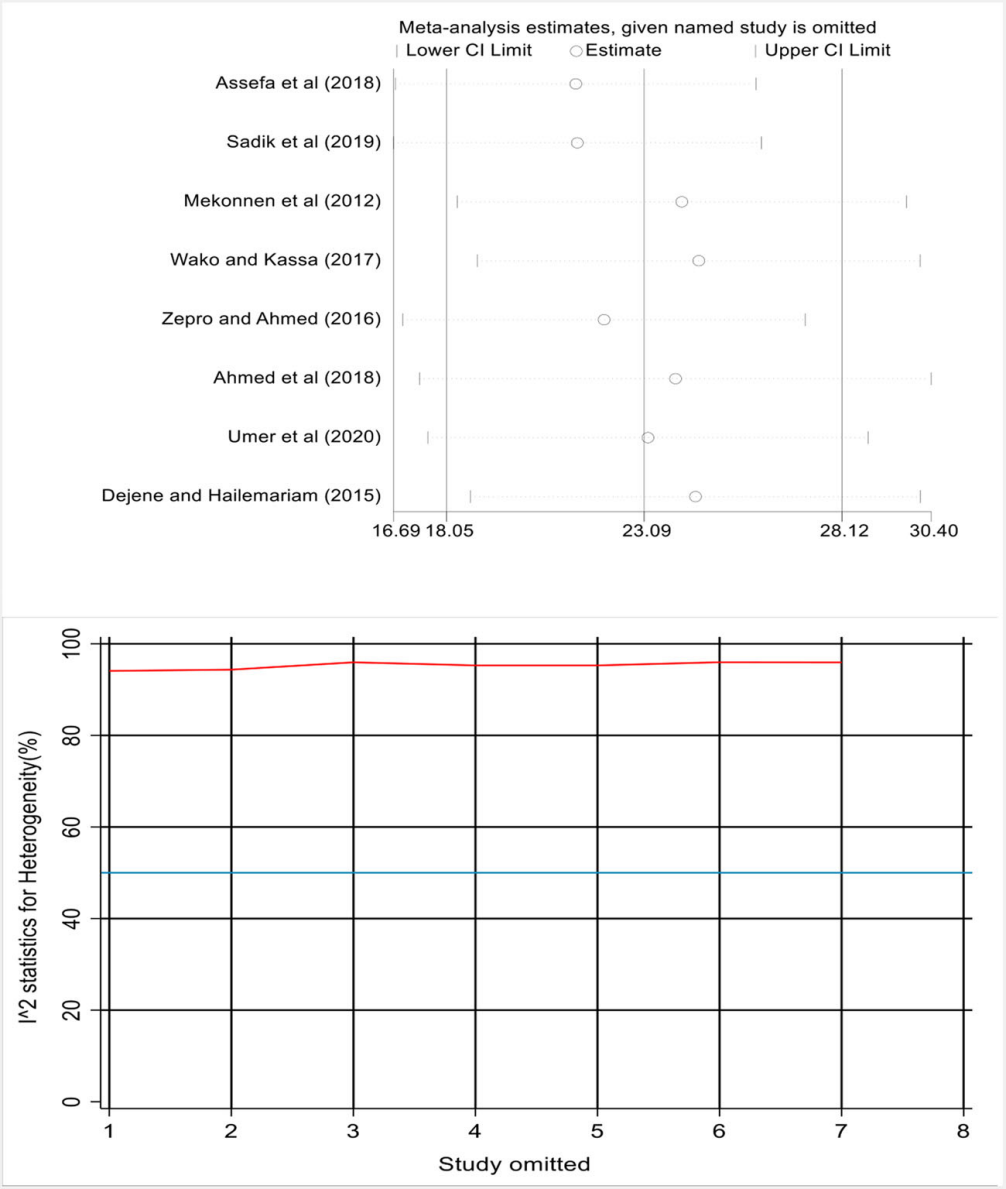
A sensitivity analysis for the pooled prevalence using the Metaninf and Metaplot commands of STATA, respectively, showed no single study had a significant effect on the pooled estimate.

The Cochrane *I* ^2^ values for this meta‐analysis were 95.36%, *p* < 0.001, suggesting the presence of noticeably high heterogeneity. Subgroup and sensitivity analyses were performed to further investigate the evidence of heterogeneity. Based on a subgroup analysis using study regions, the prevalence of health facility delivery service utilization was high in Somali region 26.40% (95% CI: 18.76−34.05, *I* ^2^ = 84.39, *p* < 0.001) and low in Oromia region 13.90% with 95% CI: 11.49−16.31. A leave‐one‐out sensitivity analysis was also executed using both metaninf and metaplot command to investigate the influence of a single study on the overall magnitude estimate and suggesting that the finding was not unduly influenced by a single study. Thus, the point estimate of its omitted analysis lies within the CI of the combined analysis and the overall heterogeneity was not significantly changed by excluding a particular study (Figure 3).

**Figure 4:**
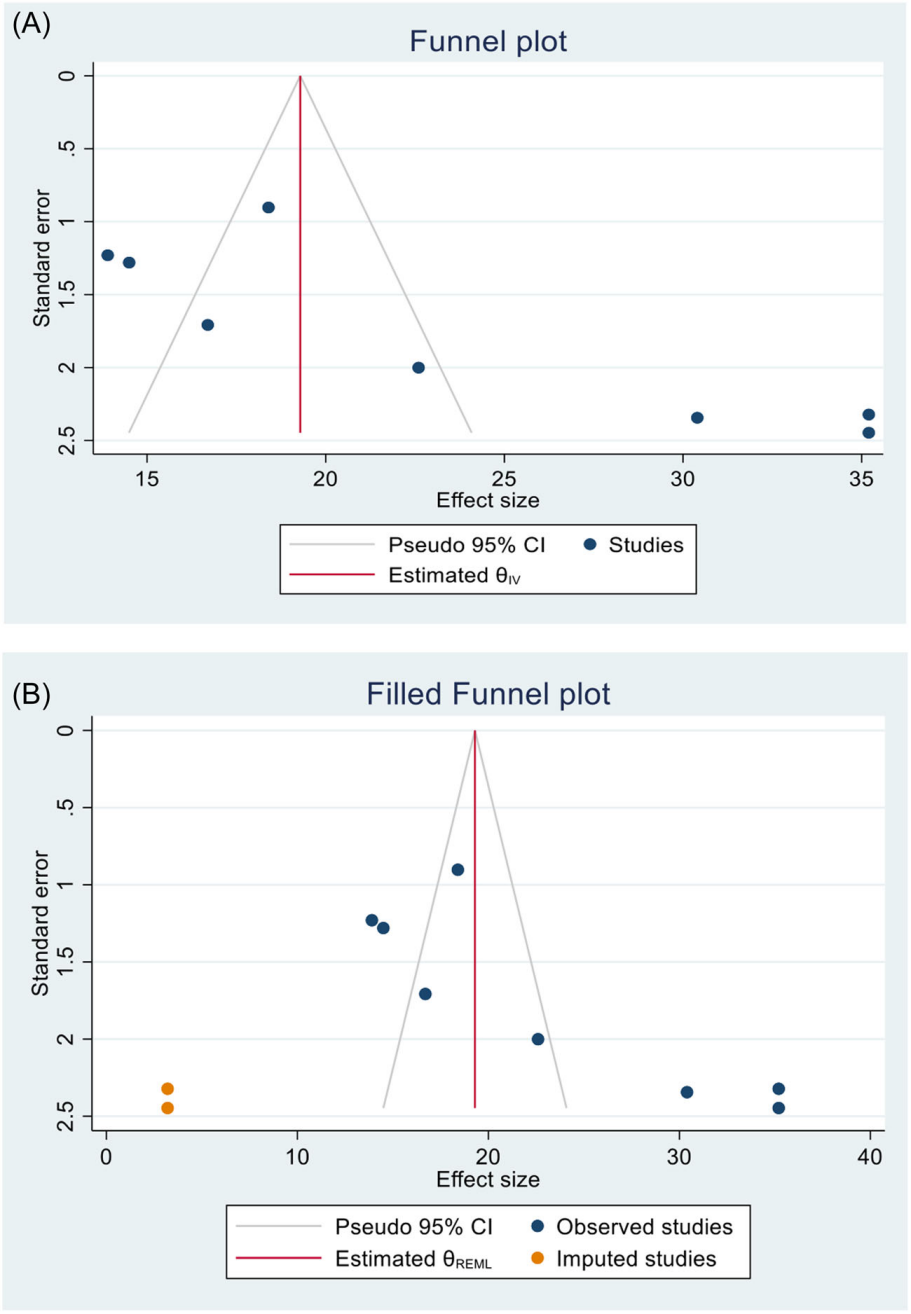
(A) A funnel plot with a pseudo 95% confidence limit used to test for publication bias. (B) A funnel plot with a pseudo 95% confidence limit after a trim‐and‐fill analysis in which two studies have been imputed.

The presence of publication bias was examined using funnel plots and tests (Egger and Begg). A funnel plot showed an asymmetrical distribution (Figure 4A,B). Besides, the results of the Egger and Begg tests showed significant evidence of publication bias (*p* < 0.05). Therefore, trim and fill analysis was conducted. After two studies were imputed using run R0 estimator for the number of missing study, the trim and fill analysis gave a pooled prevalence of 19.21 (95% CI: 13.93−24.50).

### 3.4 Factors associated with health facility delivery service utilization

Factors such as women's level of education, ANC follow‐up during pregnancy, knowledge about obstetric complications & health facility delivery service utilization, husband involvement in decision of delivery place, having information on maternal health service fee exemption, and distance to a health facility were assessed for their associations with the health facility delivery service utilization among women in pastoralist regions of Ethiopia. Of course, all factors, with the exception of the husband's involvement in the delivery location decision, have a significant impact on the health facility delivery service utilization practice.

Accordingly, women who had ANC follow‐up during pregnancy were 3.75 times (OR = 3.75, [95% CI: 1.84−7.63]) more likely to give birth at health facility than their counterpart. The odds of health facility delivery service utilization were 3.86 (OR = 3.86, [95% CI: 1.54−9.65]) times more likely among women who have good knowledge about obstetric complication & health facility delivery service utilization than their counterpart. Furthermore, the likelihood of giving birth at health facility were 9.51 times (OR = 9.51, [95% CI: 1.41−64.26]) higher among women who have information on maternal health service free exemption than those who have no information. Women who have a nearby health facility were also 3.49 (OR = 3.49, [95% CI: 1.48−8.20]) times more likely to give birth at health facility than their counterpart. The probability of health facility delivery service utilization were also 3.06 (OR = 3.06, [95% CI: 1.77−5.29]) times greater among women who attend secondary and above education than their counterpart (Table 2).

**Table 2:** Factors associated with health facility delivery service utilization among women in pastoralist regions of Ethiopia.

Determinants	Comparison	Number of studies	Total sample	Pooled OR with 95% CI	*I* ^2^ (%)	*p* Value
Women's educational status	Secondary+ (vs. below secondary)	3	2700	3.06 (1.77−5.29)	69.2	0.039
ANC follow‐up	Yes (vs. no)	8	4993	3.75 (1.84−7.63)	93.0	<0.001
Distance to health facility	Nearby (vs. far)	4	3031	3.49 (1.48−8.20)	93.7	<0.001
Information about delivery service fee	Have info (vs. not have info)	2	1193	9.51 (1.41−64.26)	95.7	<0.001
Knowledge about obstetric complication & importance of delivery service utilization.	Have good knowledge (vs. have no)	4	1852	3.86 (1.54−9.65)	92.7	<0.001

## 4 DISCUSSION

This systematic review and meta‐analysis is conducted to estimate the best available evidence for the prevalence and associated factors of health facility delivery service utilization in pastoralist regions of Ethiopia. As far as is known, no previous systematic reviews/meta‐analyses were conducted to address this issue. Accordingly, the finding indicated that pooled prevalence of health facility delivery service utilization in pastoralist regions of Ethiopia was 23.09% (95% CI: 18.05%−28.12%). Despite efforts to achieve the World Health Organization's recommendation that every pregnant woman give birth at a health facility with trained birth attendants, the rate in Ethiopia is still low, especially among pastoralist regions. This implies that the Ethiopian government is expected to do more in this area to increase the use of institutional delivery services and improve maternal and child health in pastoralist regions and the country at large.

This study's finding were much lower than those of a study based on the 2016 Ethiopia Demographic and Health Survey (32.8%)^28^ and the 2019 mEDHS national figure of health facility delivery service utilization (48%).^29^ It is also lower than a meta‐analysis and systematic review result (31%) of health facility delivery service utilization in Ethiopia.^30^ The possible explanation for this variation will be the difference in the study population and poor access to health services in pastoralist regions of Ethiopia. Furthermore, this could be due to the fact that this systematic review and meta‐analysis focused solely on Ethiopian pastoral communities, which may have limited access to the media. As a result, knowledge of obstetric complications and health facility delivery services may be limited, leading women in pastoralist regions to have home birth.

This study demonstrated that there were a significant association between health facility delivery service utilization and ANC follow‐up. Women who have ANC visit during her pregnancy were 3.75 times more likely to give birth at health facility than their counterparts. This finding is consistent with a systematic review from Ethiopia,^30^, ^31^ and primary studies conducted in Kenya^32^ & Eretria.^33^ This could be because of ANC is the most convenient way for mothers to learn more about pregnancy and delivery. Consequently, women who had no ANC visits may be less aware of birth preparedness and complication readiness plans, pregnancy danger signs, and the dangers of giving birth at home, which may make them to give childbirth at home. Furthermore, this study also revealed that women with poor knowledge of obstetric complications and health facility delivery service utilization, as well as had no information on maternal health service free exemption, were less likely to give birth in a health facility.

As this study indicates, women who had access to a nearby health facility were also 3.49 times more likely to give birth in health facility than women who did not have access to a nearby health facility. This might be due to women who reside in nearby health facilities will have no problem with transportation to attend health facility delivery at any time. Since there may be unpredictable labor and a lack of support from their spouse due to seasonal migration out of their house to feed and get water for their cattle in pastoralist regions, women who do not have access to a nearby health facility may have difficulty having a health facility delivery. Besides, women resided to the nearby health facility might have different access of maternal health services such as health education and ANC services.

The educational status of women was also found to be a significant predictor of their health facility delivery service utilization practice in this study. Women with a secondary and above education had higher odds of giving birth in health facility than those with no formal education. This is in line with studies from Uganda,^34^ Bangladesh,^35^ and Haiti.^36^ The reason behind might be educated women are more aware of the risks connected with home delivery and have a better understanding of service availability. Furthermore, learned women may be more concerned for their health & have a good decision‐making capacity making them more likely to give birth at health facility.

Significant heterogeneity and publication bias had been reported in this study. Nevertheless, the sensitivity analysis in both Metaninf and Metaplot command showed that no single study had a significant effect on the pooled prevalence. In addition, studies indicated that a high *I* ^2^ value is not always synonymous with high heterogeneity, and, like this study, when only a small number of studies with no true heterogeneity are included in the meta‐analysis, *I* ^2^ will overestimate heterogeneity.^37^, ^38^ It could not be therefore difficult to utilize this paper for different purposes. The publication bias has also been overcome by conducting trim and fill analysis. Hence, the pooled prevalence of 19.21% that resulted from the trim and fill analysis can also be considered.

## 5 LIMITATIONS OF THE STUDY

It is important to interpret the review's findings by considering the following limitations. This systematic review and meta‐analysis includes almost exclusively cross‐sectional articles. The factors and the outcome variables cannot therefore be linked to one another in time. Furthermore, as a result of the included studies' differing classifications of the variables and some studies did not provide them at all, the pooled estimates for factors related to health facility delivery service utilization were not estimated. Another limitation of this study is that it only included articles written in English, leaving out studies written in other languages.

## 6 CONCLUSION AND RECOMMENDATION

Though WHO recommends a community without home deliveries and Ethiopia has implemented numerous initiatives to attain this goal, health facility delivery service utilization is still very low in pastoralist regions of Ethiopia. In pastoralist regions of Ethiopia, factors such as having no formal education, no nearby health facility, lack of information on maternal health service free exemption, not pursuing ANC visits at all, and poor knowledge of obstetric complications and health facility delivery service utilization significantly decrease the probability of health facility delivery service utilization. Therefore, strengthening ANC services, introducing free health services to the community, and constructing health facilities for the nearby residents of the community could improve health facility delivery service utilization. More effort is also needed to ameliorate the awareness of mothers regarding obstetric complication and health facility delivery service utilization. Promoting maternal education will also be helpful. At all levels, awareness of the importance of maternal healthcare must be raised.

## AUTHOR CONTRIBUTIONS

**Anteneh Mengist Dessie**: conceptualization; data curation; formal analysis; methodology; writing—original draft; writing—review & editing. **Denekew Tenaw Anley**: Formal analysis; writing—original draft; writing—review & editing. **Melkamu Aderajew Zemene**: Conceptualization; data curation; resources; writing—original draft; writing—review & editing. **Eden Workneh Aychew**: Conceptualization; data curation; supervision; writing—original draft; writing—review & editing. **Habtamu Geremew Debebe**: Conceptualization; data curation; investigation; visualization; writing—original draft; writing—review & editing. **Natnael Moges Misganaw**: Investigation; methodology; supervision; writing—review & editing. **Chalachew Yenew Denku**: Conceptualization; data curation; methodology; writing—review & editing. **Tiruayehu Getinet Abebe**: Conceptualization; data curation; software; writing—original draft; writing—review & editing. **Asaye Alamneh Gebeyehu**: Conceptualization; data curation; formal analysis; writing—original draft; writing—review & editing. **Desalegn Tesfa Asnakew**: Conceptualization; formal analysis; resources; writing—review & editing. **Rahel Mulatie Anteneh**: Investigation; methodology; resources; writing—review & editing. **Sefineh Fenta Feleke**: Conceptualization; data curation; software; visualization; writing—review & editing.

## CONFLICT OF INTEREST STATEMENT

The authors declare no conflict of interest.

## TRANSPARENCY STATEMENT

The lead author Anteneh Mengist Dessie affirms that this manuscript is an honest, accurate, and transparent account of the study being reported; that no important aspects of the study have been omitted; and that any discrepancies from the study as planned (and, if relevant, registered) have been explained.

## Data Availability

Data will be available from the corresponding author with a reasonable request.
